# Total hip arthroplasty in patients with alkaptonuria: a case report

**DOI:** 10.3389/fsurg.2026.1821267

**Published:** 2026-08-31

**Authors:** Bo Zhu, Pengbo Ruan, Zicheng Hu, Han Ding

**Affiliations:** 1 Tianjin Medical University General Hospital Airport Hospital, Tianjin, China; 2 Second Clinical Medical College of Tianjin Medical University, Tianjin, China

**Keywords:** Alkaptonuria (AKU), Direct anterior approach, HGD gene, Total hip arthroplasty (THA), Variant of uncertain significance (VUS)

## Abstract

Alkaptonuria (AKU) is a rare autosomal recessive disorder caused by mutations in the homogentisate 1,2-dioxygenase (HGD) gene, often leading to debilitating ochronotic arthropathy that requires total joint arthroplasty (THA). This case report describes a 56-year-old male patient with AKU who underwent THA due to end-stage arthropathy. Genetic testing revealed that the subject carried a c.157C > T (p.Arg53Trp) variant, a missense variant in the coding region of the HGD gene, and a c.880-9T > A variant, a single base substitution in the intronic region of the HGD gene. Both were classified as variants of uncertain significance (VUS). The preoperative Visual Analog Scale (VAS) score was 4, which decreased to 2 by postoperative day 5 and reached 0 at the 1-month follow-up. At six months postoperatively, the patient reported no pain, free hip range of motion, and high satisfaction. For patients with ochronotic end-stage arthropathy, arthroplasty can achieve favorable early clinical outcomes, although longer follow-up is needed.

## Introduction

Alkaptonuria (AKU) is a rare autosomal recessive inherited metabolic disorder caused by deletions or mutations in the HGD gene located on the long arm of human chromosome 3 (1, 2). The global incidence of AKU is approximately 1 in 1,000,000 to 1 in 250,000 (3), though it is significantly higher in certain countries such as Slovakia, where the incidence reaches 1 in 19,000 (4). Since the first case of AKU was reported in China in 1959, fewer than 100 cases have been documented to date (5). The classic triad of AKU includes homogentisic aciduria, blue-black pigmentation of connective tissues, and ochronotic arthritis. Treatment options for AKU are limited (6), with conservative management aimed at alleviating symptoms and controlling disease progression (7).

Ochronotic arthropathy is a rare indication for THA. Most cases reported in the literature have adopted the posterior or posterolateral approach to achieve a wider surgical field for addressing potential severe anatomical deformities and fragile soft tissues. The direct anterior approach (DAA) for hip arthroplasty theoretically avoids muscle, nerve, and vascular injury by utilizing an intermuscular and internervous plane, aligning with the goals of minimally invasive surgery (8, 9). Compared to the posterior approach, DAA shows clear advantages in terms of average hospital stay, severity of postoperative pain, and early functional recovery (10, 11). However, reports on the early safety and efficacy of the DAA in this specific population with ochronosis are extremely limited.

This case report presents a 56-year-old male patient with AKU, who was diagnosed with compound heterozygous missense mutations in the HGD gene through whole-exome sequencing. The patient underwent DAA-THA due to end-stage ochronotic arthritis. Although most existing evidence for DAA derives from routine osteoarthritis populations, its application in rare bone disorders such as AKU remains underexplored. This case report aims to: (1) describe the technical feasibility and perioperative management of DAA-THA in this rare metabolic osteoarthropathy; (2) report two rare HGD variants (including a novel intronic variant c.880-9T > A) classified as VUS; (3) highlight the value of detailed clinical phenotyping for future genotype-phenotype-surgical outcome correlation studies in AKU. All studies were conducted after obtaining signed informed consent.

## Case report

**Figure 1:**
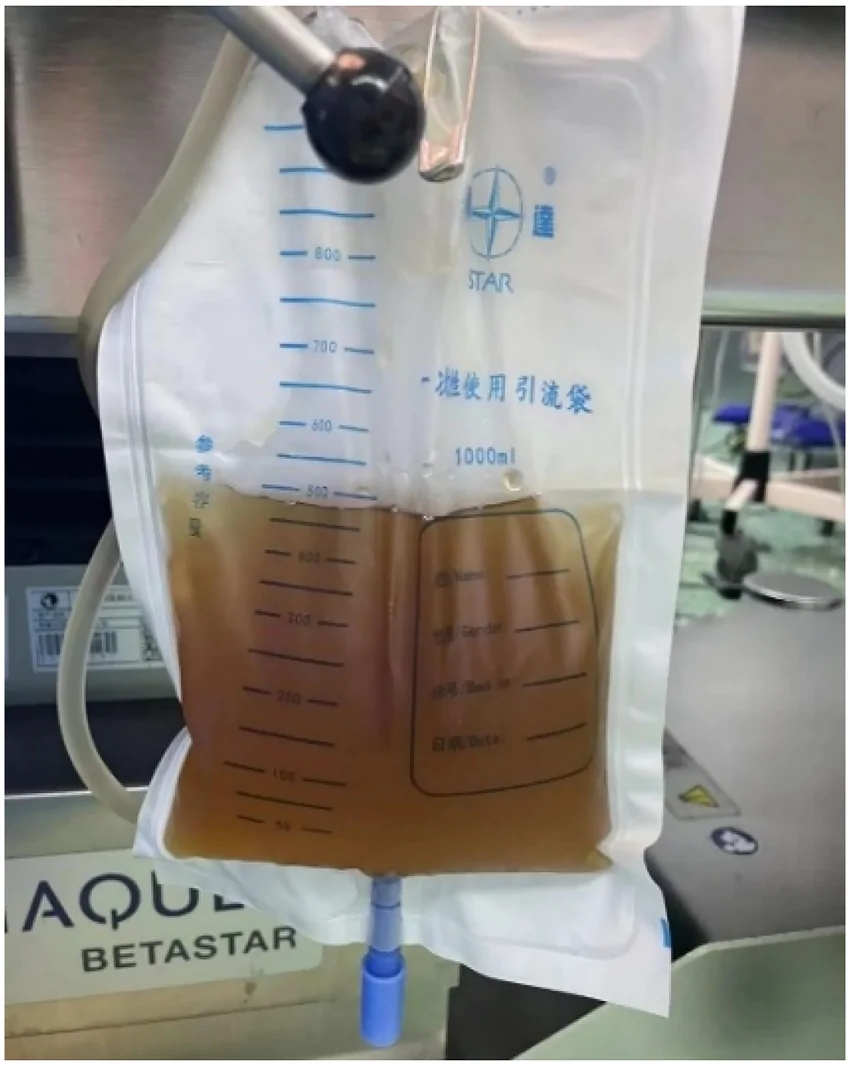
Patient urine sample, after standing.

**Figure 2:**
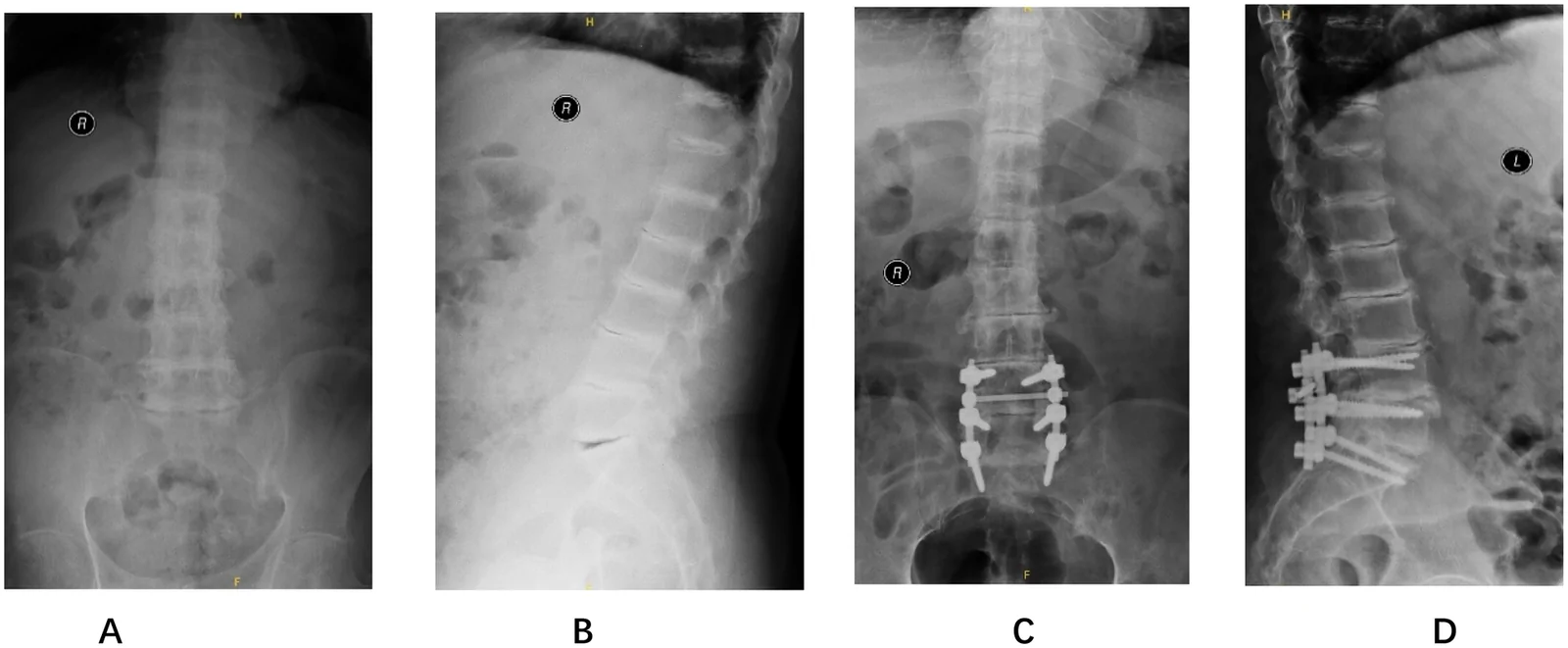
**(A,B)** Preoperative x-ray revealed loss of lumbar physiological lordosis with concomitant kyphosis, multilevel disc calcification, and vacuum phenomenon. **(C,D)** Postoperative anteroposterior and lateral x-ray.

The patient is a 56-year-old male who presented with bilateral hip pain accompanied by functional impairment. The symptoms were exacerbated by activity and alleviated with rest, with recurrent episodes. Symptoms improved following treatment with nonsteroidal anti-inflammatory drugs. Additionally, there was weakness in hip flexion and extension, more pronounced on the right side. The patient's admission blood routine test indicated microcytic hypochromic anemia, and urine biochemical test suggests significantly elevated homogentisic acid levels. Echocardiography revealed aortic valve calcification and mild mitral and tricuspid regurgitation. Lower extremity vascular ultrasound indicated patency of blood flow in both lower limbs without thrombosis. Urinary ultrasound examination suggests prostatic hyperplasia with multiple calcified plaques. Thyroid ultrasound suggests multiple hypoechoic nodules in the left lobe of the thyroid. Since childhood, the patient's urine has turned black after standing, and the urine color is dark (see Figure 1). The patient has a history of calcium pyrophosphate crystal deposition disease for over 4 years, treated with oral nonsteroidal anti-inflammatory drugs; a history of reflux esophagitis for 4 years, treated symptomatically; and a history of iron deficiency anemia, treated with oral polysaccharide-iron complex capsules. In 2016, the patient underwent cervical fusion surgery for cervical spinal stenosis (specific surgical procedure unknown). Four years ago, the patient underwent posterior lumbar decompression with internal fixation (L4–S1) and bone graft fusion (L5/S1) at our hospital due to lumbar spinal stenosis. Preoperative and postoperative imaging studies of the spine are shown in Figures 2A–D. The patient's parents are not consanguineous, and there is no family history of genetic diseases. Physical examination of the hips indicated pain during bilateral hip movement, more significant on the right side. Tenderness was noted over the greater trochanter region, with bilateral hip adduction deformity. The Patrick test was positive. Imaging findings demonstrated bilateral femoral head necrosis, secondary to degenerative osteoarthropathy of both hips.

**Figure 3:**
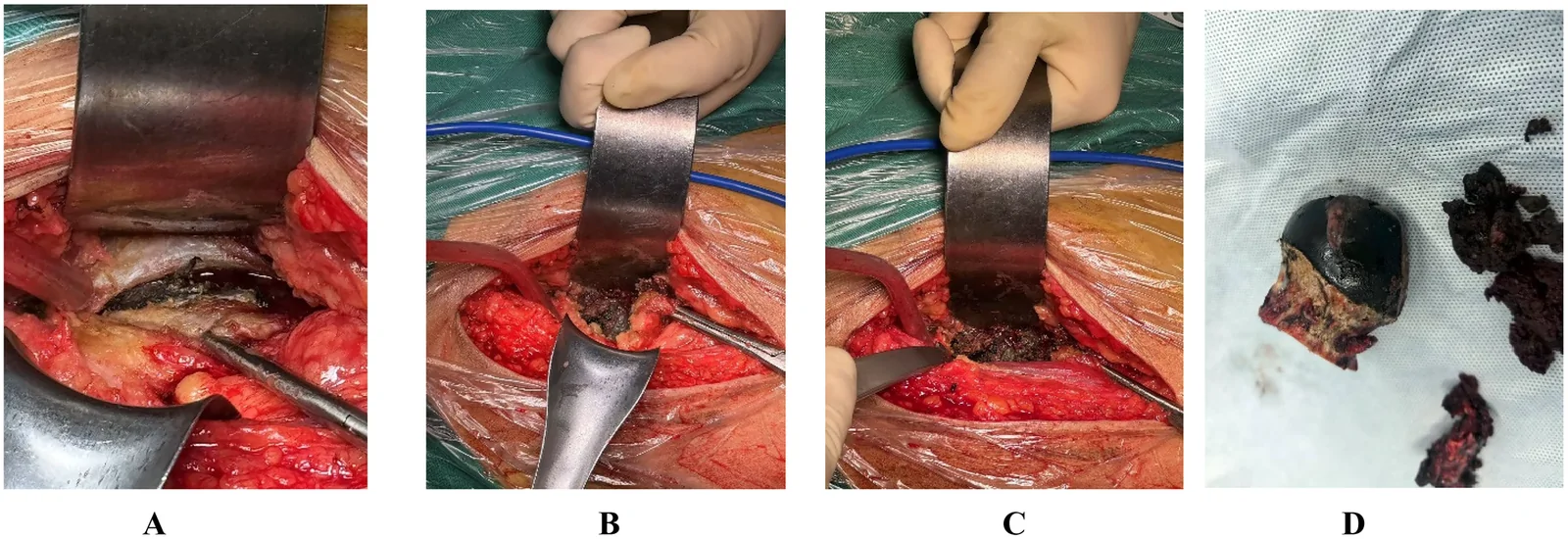
**(A,B)** Extensive dark brown discoloration of the synovial tissue around the hip joint, with fibrotic and contracted hip joint capsule presenting blackened changes. **(B)** Place the hohmann retractor in the femoral neck saddle area to expose the hip joint. **(C)** Place the hohmann retractor in the femoral neck saddle area to expose the hip joint. **(D)** The surface cartilage of the femoral head exhibits extensive dark brown pigmentation, resembling the appearance of a “mangosteen”.

Based on the patient's laboratory findings and physical examination results, the diagnoses of rheumatoid arthritis and ankylosing spondylitis were ruled out preoperatively. After completing the preoperative evaluation, the patient underwent right-sided THA on the fourth day after admission, as the symptoms on that side were more severe. Routine preoperative surgical informed consent was obtained. The patient was placed in the supine position with the hip elevated to facilitate exposure of the femoral side. Intraoperative findings revealed extensive dark brown discoloration of the synovial tissue surrounding the hip joint (Figures 3A,B). The hip joint capsule exhibited fibrotic contracture with blackish changes (Figures 3C). The joint was exposed after resection of the joint capsule. The femoral neck was osteotomized above the lesser trochanter, and the femoral head was removed intact. The surface of the femoral head showed widespread dark brown pigmentation of the cartilage, resembling the appearance of a “mangosteen” (Figures 3D). The acetabular labrum and surrounding osteophytes were removed. Acetabular bone preparation proved challenging due to sclerotic changes in the acetabular bone, which was hard and difficult to ream. Sequential careful reaming was performed starting with small-sized reamers to expose the subchondral bone. Significant osteoporotic changes were observed in the subchondral bone. Reaming continued until widespread, uniform bleeding was achieved from the acetabulum. A cementless acetabular cup (size 54 mm outer diameter, Tianjin Zhengtian Medical Equipment Co., Ltd.) with a highly cross-linked polyethylene liner was implanted after achieving uniform subchondral bleeding. A cementless femoral stem (size 5, Tianjin Zhengtian Medical Equipment Co., Ltd.) with a ceramic femoral head (diameter 36 mm) was used. No cement was used for either component. The operation lasted approximately 1.5 h, with an intraoperative blood loss of approximately 200 mL. The wound was irrigated and closed in layers.

**Figure 4:**
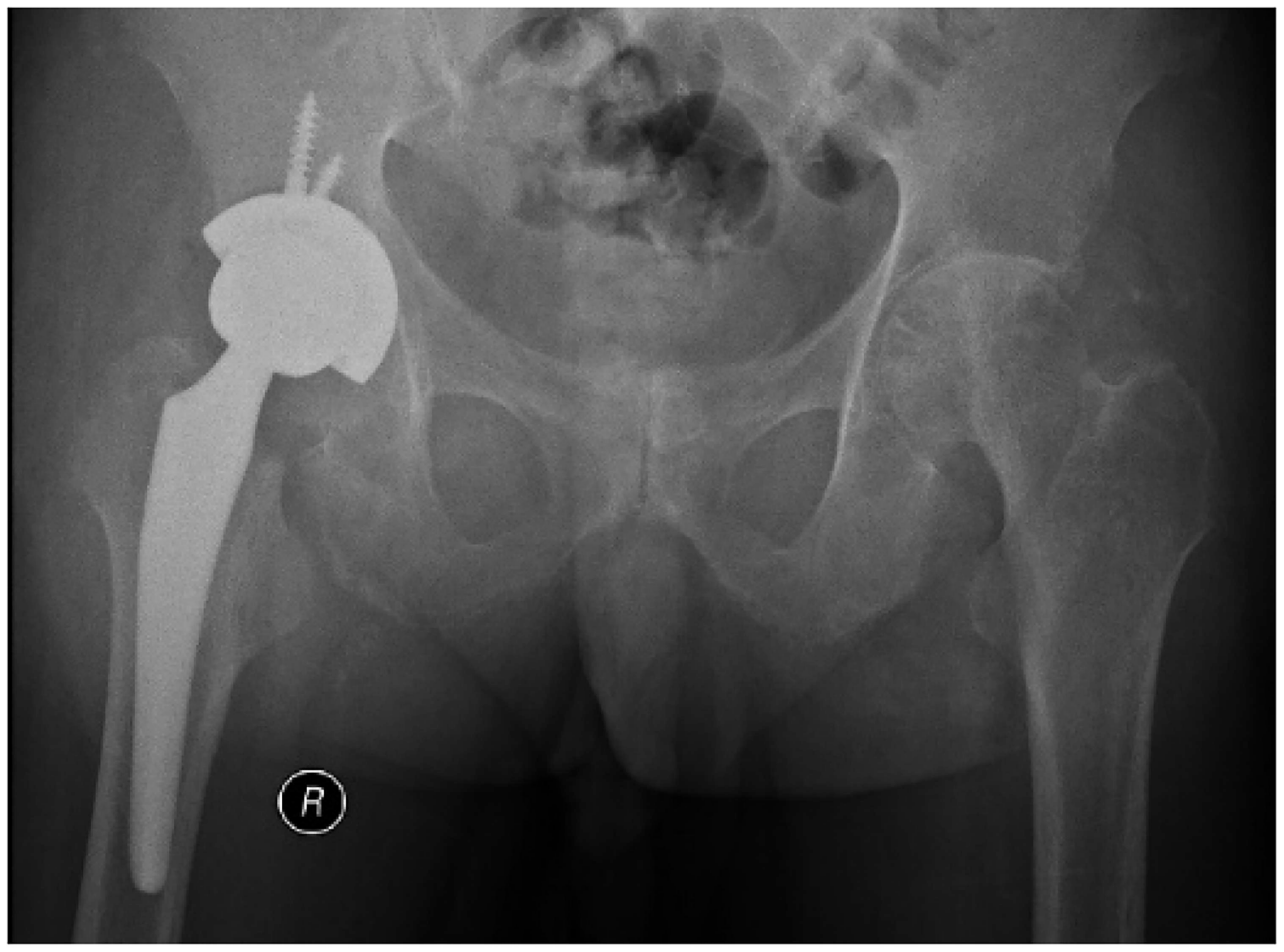
Postoperative anteroposterior radiograph of the hip (Tianjin zhengtian medical equipment Co., Ltd.).

The patient was able to ambulate with full weight-bearing on the first postoperative day. The patient received standard rehabilitation training in the hospital after surgery and was instructed to conduct self-rehabilitation training at home after discharge. Postoperative imaging evaluation revealed satisfactory positioning of the prosthetic components (Figure 4). Histopathological examination of the resected bone and residual soft tissue during surgery showed fibrous tissue hyperplasia with inflammatory cell infiltration, scattered pigment deposition, multinucleated giant cell reaction, and localized granuloma formation, all of which are characteristic manifestations of ochronosis (Figure 3). The postoperative genetic test results are shown in Table 1. Based on current evidence, these variants are classified as variants of uncertain clinical significance. Pain visual analog scale (VAS) score (0–10, 0 indicating no pain, 10 indicating the intolerable pain) were evaluated prior to operation, then the postoperative assessments of pain VAS score were performed on day 1, day 3, day 5, at month 1 and month 6, respectively. The preoperative VAS score was 4 points, the first postoperative day was 3 points, decreased to 2 points on the 3rd and 5th postoperative days, and at the 1-month follow-up, the VAS score decreased to 0 points. At the six-month postoperative follow-up, the patient reported no pain, exhibited free range of motion in the hip joint, and expressed high satisfaction with the surgical outcome. The patient attended all scheduled follow-up visits and adhered to the home-based rehabilitation program as instructed. The patient's left hip remained symptomatic but was managed conservatively during the 6-month follow-up. A staged left THA will be considered if pain progresses or functional status deteriorates.

**Figure 5:**
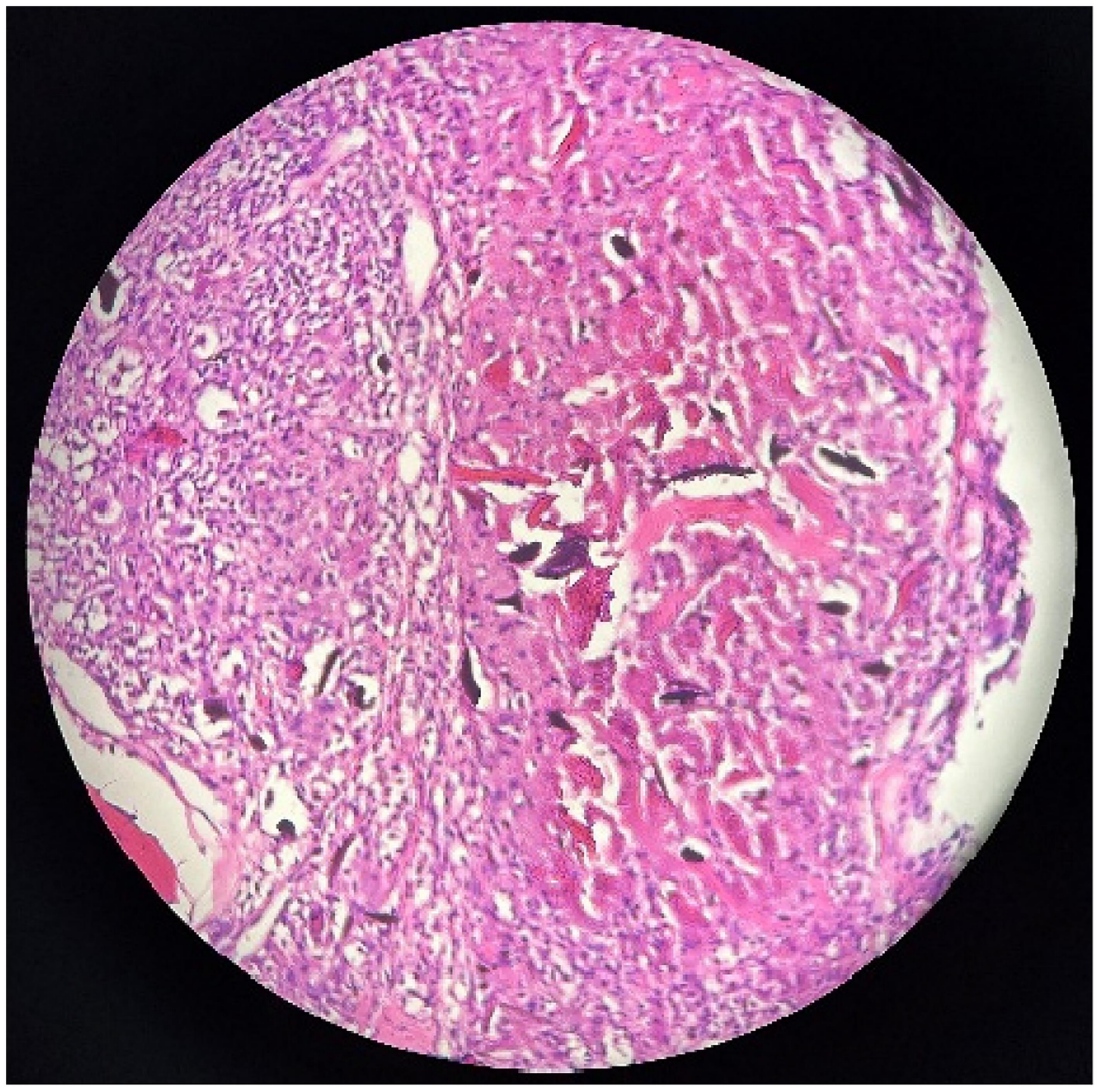
The pathological findings revealed brown-yellow pigmentation in the medullary cavity and a multinucleated giant cell reaction (H&E staining, original magnification, ×200).

**Table 1:** Molecular test report.

Gene	Chromosomal location	Mutation information	Zygosity type	Disease name	Variant origin	Variant classification
HGD	chr3:120393767	NM_000187.4:c.157C > T(p.Arg53Trp)	hybrid	AKU	NA	VUS
HGD	chr3:120357437	NM_000187.4:c.880- 9T > A	hybrid	AKU	NA	VUS

HGD, homogentisic acid dioxygenase; AKU, Alkaptonuria; NA, not available; VUS, variant of uncertain significance.

## Discussion

The diagnosis of AKU was initially delayed because the patient's initial symptoms were non-specific. Genetic testing was performed postoperatively, and the identification of two VUS added uncertainty, which was resolved by correlating with typical clinical and intraoperative findings. Ochronosis is the most common complication of AKU (12). In ochronotic arthritis, physical therapy, pain control, and nonsteroidal anti-inflammatory drugs can alleviate symptoms but have no effect on disease progression (13). Nitisinone, a competitive inhibitor of 4-hydroxyphenylpyruvate dioxygenase, reduces homogentisic acid production and is approved for the treatment of AKU in some countries. However, its effect on established ochronotic arthropathy is limited, and it is not a substitute for arthroplasty in end-stage joint destruction. The patient in this case was not on nitisinone at presentation or during follow-up. In end-stage cases of ochronotic arthropathy, total joint arthroplasty represents the best treatment option (7, 14). Combined with early clinical improvement and radiographic results, the DAA approach is a technically feasible option in cases of ochronotic arthropathy, but further promotion and application require accumulation of more cases.

The c.157C > T (p.Arg53Trp) missense variant in the HGD gene is rarely reported in gnomAD or AKU-related literature (15, 16). The c.880-9T > A intronic variant has not been documented in any published study or population database, representing the first reported case. According to the ACMG/AMP guidelines for sequence variant interpretation (17), both variants were classified as “Variants of Uncertain Significance” (VUS).

This case has the following limitations: First, although two HGD gene variants (c.157C > T and c.880-9T > A) were identified by next-generation sequencing, they were not confirmed by Sanger sequencing, posing a theoretical risk of false-positive results (18). The c.880-9T > A variant, as an intronic mutation, lacks functional validation at the RNA level to confirm its impact on splicing (19). Secondly, the failure to obtain family samples for co-segregation analysis prevented confirmation of the two variants being in trans configuration, which constitutes the missing key evidence for establishing compound heterozygous VUS as the pathogenic cause (17, 20). Future work includes *in vitro* functional validation and data sharing registration.

## Conclusion

This case report illustrates the technical feasibility of the direct anterior approach for total hip arthroplasty in a patient with AKU-associated hip arthropathy, with no early postoperative complications. Regarding the genetic findings, the patient carries two rare HGD gene variants (c.157C > T and c.880-9T > A). However, both are classified as VUS, and thus, without additional functional studies or family co-segregation analysis, the exact causal relationship between these specific variants and the clinical phenotype cannot be determined. This report adds two rare variants to the AKU mutation spectrum and underscores the value of detailed clinical phenotyping for future VUS reclassification. Longer follow-up and accumulation of similar cases are required.

## Data Availability

The original contributions presented in the study are included in the article/Supplementary Material, further inquiries can be directed to the corresponding author.
